# Multisite phosphorylation of C-Nap1 releases it from Cep135 to trigger centrosome disjunction

**DOI:** 10.1242/jcs.142331

**Published:** 2014-06-01

**Authors:** Tara Hardy, Miseon Lee, Rebecca S. Hames, Suzanna L. Prosser, Donna-Marie Cheary, Mugdha D. Samant, Francisca Schultz, Joanne E. Baxter, Kunsoo Rhee, Andrew M. Fry

**Affiliations:** 1 Department of Biochemistry, University of Leicester, Lancaster Road, Leicester LE1 9HN, UK; 2 Department of Biological Sciences, Seoul National University, Seoul 151-747, Republic of Korea

**Keywords:** Centrosome disjunction, Centriolar linker, C-Nap1, Cep135, Nek2

## Abstract

During mitotic entry, centrosomes separate to establish the bipolar spindle. Delays in centrosome separation can perturb chromosome segregation and promote genetic instability. However, interphase centrosomes are physically tethered by a proteinaceous linker composed of C-Nap1 (also known as CEP250) and the filamentous protein rootletin. Linker disassembly occurs at the onset of mitosis in a process known as centrosome disjunction and is triggered by the Nek2-dependent phosphorylation of C-Nap1. However, the mechanistic consequences of C-Nap1 phosphorylation are unknown. Here, we demonstrate that Nek2 phosphorylates multiple residues within the C-terminal domain of C-Nap1 and, collectively, these phosphorylation events lead to loss of oligomerization and centrosome association. Mutations in non-phosphorylatable residues that make the domain more acidic are sufficient to release C-Nap1 from the centrosome, suggesting that it is an increase in overall negative charge that is required for this process. Importantly, phosphorylation of C-Nap1 also perturbs interaction with the core centriolar protein, Cep135, and interaction of endogenous C-Nap1 and Cep135 proteins is specifically lost in mitosis. We therefore propose that multisite phosphorylation of C-Nap1 by Nek2 perturbs both oligomerization and Cep135 interaction, and this precipitates centrosome disjunction at the onset of mitosis.

## INTRODUCTION

Centrosomes are the primary microtubule-organizing centers in animal cells. They are composed of centrioles, short cylinders of nine triplets of highly stabilized microtubules arranged into a pinwheel configuration and surrounded by pericentriolar material (PCM) from which microtubules are nucleated (Bornens, 2012; Doxsey et al., 2005). Centrosomes are usually observed experimentally by using markers of the PCM and, on this basis, normal cells possess two centrosomes throughout the cell cycle. In G1, each centrosome has a single centriole that is a polarized structure with a proximal and distal end. However, the two centrioles that are present in G1 are not identical; the older mother centriole has distal and sub-distal appendages, which the younger daughter centriole lacks. As cells progress into S and G2, each centriole is duplicated, with a new centriole, known as a pro-centriole, growing in close proximity to the proximal end of both mother and daughter centrioles. As cells then pass through mitosis, the daughter centriole acquires appendages and matures into a mother, whereas the two pro-centrioles mature into the new daughters of the offspring cells. Importantly, many cancer cells possess more than two centrosomes, and considerable effort is now being put into understanding the mechanisms that lead to the generation of these supernumerary or amplified centrosomes (Bettencourt-Dias and Glover, 2007; Nigg and Stearns, 2011).

Owing to their organization, centrosomes in G2 cells undergo two distinct separation events during mitotic progression. The first occurs in late G2 or early M, when the two centrosomes, each now containing a pair of centrioles, move apart to establish the opposing poles of the mitotic spindle. The second separation event occurs as cells exit mitosis, when the pair of centrioles within each pole splits apart or ‘disengages’, enabling each daughter cell to inherit two centrioles, which are now separated to form the two centrosomes. However, although progress has been made into understanding the biochemical regulation of these events (Mardin and Schiebel, 2012; Tanenbaum and Medema, 2010), the mechanistic details are lacking.

Both before and after centriole duplication, the two centrosomes generally remain in close proximity throughout interphase. In part, this is due to the action of the microtubule and actin cytoskeletons, which push against the cell cortex to keep the centrosomes in the cell center (Euteneuer and Schliwa, 1985; Jean et al., 1999; Thompson et al., 2004). However, centrosomes remain as paired structures when isolated from cultured cells, and electron-dense material can be seen connecting the centrosomes (Nigg, 2002; Paintrand et al., 1992). Hence, the current view is that a physical linker connects the two centrosomes in interphase. This linker is assembled in G1 to physically tether mother and daughter centrioles after they have undergone disengagement, is maintained through S and G2 as centrioles duplicate, but then is disassembled during mitotic onset to allow centrosomes to separate and establish the mitotic spindle. The untethering of this linker is known as centrosome disjunction.

In terms of its composition, a number of linker components have been identified, including C-Nap1 (also called CEP250), rootletin, Cep68, LRRC45, centlein and β-catenin (Bahe et al., 2005; Bahmanyar et al., 2008; Fang et al., 2014; Fry et al., 1998a; Graser et al., 2007; Hadjihannas et al., 2010; He et al., 2013; Yang et al., 2006). C-Nap1 is a large (281 kDa) coiled-coil protein that localizes to the proximal ends of mother and daughter centrioles. Here, it acts as a platform for the recruitment of the related protein rootletin. As its name suggests, rootletin is a major component of the striated ciliary rootlet present in monociliated cells (Yang et al., 2002). However, in the context of dividing cells, rootletin forms entangling filaments that provide the major connecting element of the intercentriolar linker (Bahe et al., 2005). As for Cep68, LRRC45, centlein and β-catenin, their role in the linker remains to be determined, although they might contribute to the stability of the C-Nap1–rootletin assembly. Consistent with a role for C-Nap1 and rootletin in forming the core of the linker, they are present on interphase centrosomes but lost from spindle poles upon mitotic entry, and their depletion by RNA interference leads to premature loss of centrosome cohesion in interphase (Bahe et al., 2005; Graser et al., 2007).

So if these proteins constitute the linker then what regulates their disassembly at the G2/M transition? This is the role of the Nek2 protein kinase, a member of the human NIMA-related kinase family (Fry et al., 2012; Moniz et al., 2011). Nek2 physically interacts with C-Nap1 and rootletin, can phosphorylate these proteins and, when overexpressed, leads to their displacement from the centrosome and loss of centrosome cohesion (Bahe et al., 2005; Faragher and Fry, 2003; Fry et al., 1998a; Fry et al., 1998b). Nek2 also interacts with protein phosphatase 1 (PP1) through a motif in the C-terminal non-catalytic domain of Nek2. It is hypothesized that, when bound to Nek2, PP1 can dephosphorylate both Nek2 and C-Nap1, preventing the untimely separation of centrosomes during interphase (Helps et al., 2000; Meraldi and Nigg, 2001). Furthermore, binding of inhibitor-2, a physiological PP1 inhibitor, at the G2/M transition could enhance phosphorylation of C-Nap1, promoting linker disassembly and centrosome separation (Eto et al., 2002; Mi et al., 2007).

So what controls the activity of Nek2? Nek2 undergoes autophosphorylation within its activation loop in response to homodimerization through a leucine zipper present downstream of the N-terminal catalytic domain (Croasdale et al., 2011; Fry et al., 1999). However, there is no evidence to date that the dimerization or autophosphorylation activity are regulated. Nek2 is also subject to phosphorylation by an upstream kinase, Mst2 (also known as STK3) (Mardin et al., 2010). This phosphorylation, which occurs in the non-catalytic C-terminal region, increases the affinity of Nek2 for the centrosome. In turn, Mst2 is phosphorylated by the mitotic kinase Plk1, and this regulates the affinity of PP1 for the Nek2–C-Nap1 complex, putting Plk1 upstream of Nek2 (Mardin et al., 2011; Smith et al., 2011).

Experimental approaches that interfere with centrosome disjunction, such as Nek2 depletion, do not prevent bipolar spindle assembly or mitotic progression. This is because multiple processes contribute to centrosome separation, including force generation by microtubule-based motors that drive poles apart through anti-parallel microtubule sliding (Tanenbaum and Medema, 2010). However, in the absence of Nek2, this requires robust motor activity, possibly to rip the linker apart, as reduced motor activity makes bipolar spindle assembly Nek2-dependent (Mardin et al., 2010). Importantly, delayed centrosome separation is detrimental to genome integrity, as it enhances the frequency of merotelic attachments that lead to chromosome segregation errors (Indjeian and Murray, 2007; Kaseda et al., 2012; Mardin et al., 2013; Silkworth et al., 2012). Hence, defects in the Nek2 pathway that delay centrosome separation would have detrimental consequences on genome stability. Here, we have investigated the mechanistic basis for how Nek2 regulates centrosome disjunction. We show that Nek2 can phosphorylate multiple sites on the C-terminal domain (CTD) of C-Nap1 and suggest that, rather than site-specific modulation, it is a change in overall charge of this domain that perturbs oligomerization and centrosome localization. Moreover, our data support a model in which hyperphosphorylation of C-Nap1 triggers centrosome disjunction through loss of interaction with the centriole proximal end protein Cep135.

## RESULTS

### The intercentriolar linker is a stable structure that recruits Nek2

**Fig. 1.**
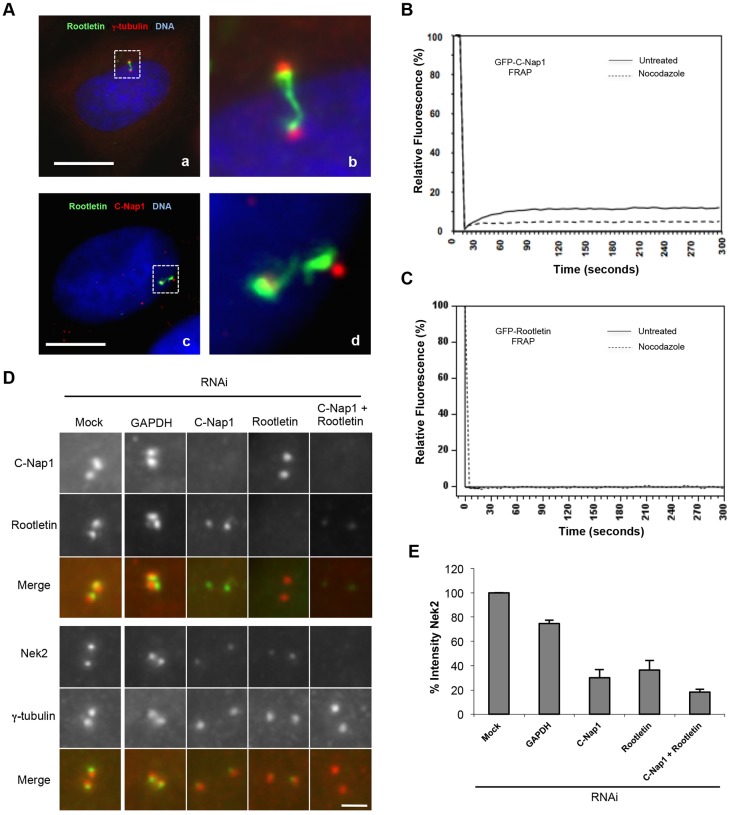
**Nek2 is recruited to the centrosome by stable intercentriolar linker components.** (A) Immunofluorescence images of HeLa cells stained with antibodies against rootletin (green) and either γ-tubulin (a,b) or C-Nap1 (c,d) (red), and Hoechst 33342 to visualize DNA (blue). Panels b and d are enlargements of the regions outlined in panels a and c, respectively. Scale bars: 10 µm. (B,C) FRAP analysis of U2OS cells transiently transfected for 24 hours with GFP-tagged C-Nap1 (B) or rootletin (C) and treated with or without nocodazole to depolymerize microtubules. A region of interest (ROI) encompassing the centrosomes was selected and three images were collected before the ROI was bleached three times (100% argon laser, 488 nm). The fluorescence intensity relative to pre-bleach was then calculated over 300 seconds for the ROI and an average recovery profile was plotted for each construct (*n* = 20). (D) Immunofluorescence images of centrosomes from HeLa cells that were either mock treated or depleted by RNAi for 48 hours of GAPDH, C-Nap1, rootletin or C-Nap1 and rootletin together, and stained with antibodies against C-Nap1 (red) and rootletin (green), or γ-tubulin (red) and Nek2 (green), as indicated. Merged images are shown. Scale bar: 1.5 µm. (E) The centrosome intensity of Nek2 in cells depleted as in D relative to mock-depleted cells. Data show the mean±s.d. (three independent experiments, 35 centrosomes per condition per experiment).

To study the mechanisms regulating centrosome disjunction, we first raised a polyclonal antibody against rootletin to confirm the presence of intercentriolar fibers in interphase cells. This antibody revealed filamentous structures that extend from each of the two centrosomes and that in some, albeit not all, cells could be seen linking the two centrosomes (Fig. 1A). These fibers were distinct from, and generally sat between, the more restricted staining of C-Nap1 that localizes to centriole proximal ends. We then examined how dynamic C-Nap1 and rootletin were at interphase centrosomes using cells expressing GFP-tagged versions of these proteins. Using a fluorescence recovery after photobleaching (FRAP) assay, we found that the bulk of C-Nap1 and rootletin remained stably associated with the centrosomes, with C-Nap1 exhibiting a small fraction (∼10%) of turnover with a half-life (t_1/2_) of ∼30 seconds, but no recovery of rootletin (Fig. 1B,C). The limited recovery of C-Nap1 was inhibited following microtubule depolymerization (Fig. 1B; supplementary material Fig. S1A). We hypothesize that most centrosomal C-Nap1 is assembled into a stable linker structure, but excess C-Nap1 trafficked to the centrosome along microtubules is released owing to the limited binding capacity at the proximal ends of centrioles.

The stability of the linker components is in stark contrast to centrosomal Nek2, which is predominantly (∼70%) dynamic with a t_1/2_ of ∼5 seconds (Hames et al., 2005). To test whether centrosomal recruitment of Nek2 depends upon interaction with the more stable components, we depleted C-Nap1 and rootletin and measured the centrosomal intensity of Nek2 by immunofluorescence microscopy (Fig. 1D,E). Depletion of rootletin or C-Nap1 alone led to a significant reduction in centrosomal Nek2, which was further reduced upon their co-depletion. Hence, we conclude that centrosome localization of Nek2 is largely dependent on dynamic association with rootletin and C-Nap1. The remaining Nek2 at the centrosome could represent the small fraction recently reported to localize to the distal end of the mother centriole (Spalluto et al., 2012). As expected, depletion of C-Nap1 and rootletin induced centrosome splitting; however, the decrease in Nek2 was similar on split and unsplit centrosomes (data not shown). Furthermore, C-Nap1 depletion led to a partial reduction in rootletin staining and less prominent rootletin fibers, consistent with the model that C-Nap1 acts as a platform for rootletin fiber assembly (Bahe et al., 2005).

### Nek2 phosphorylates multiple sites within the C-Nap1 CTD

**Fig. 2.**
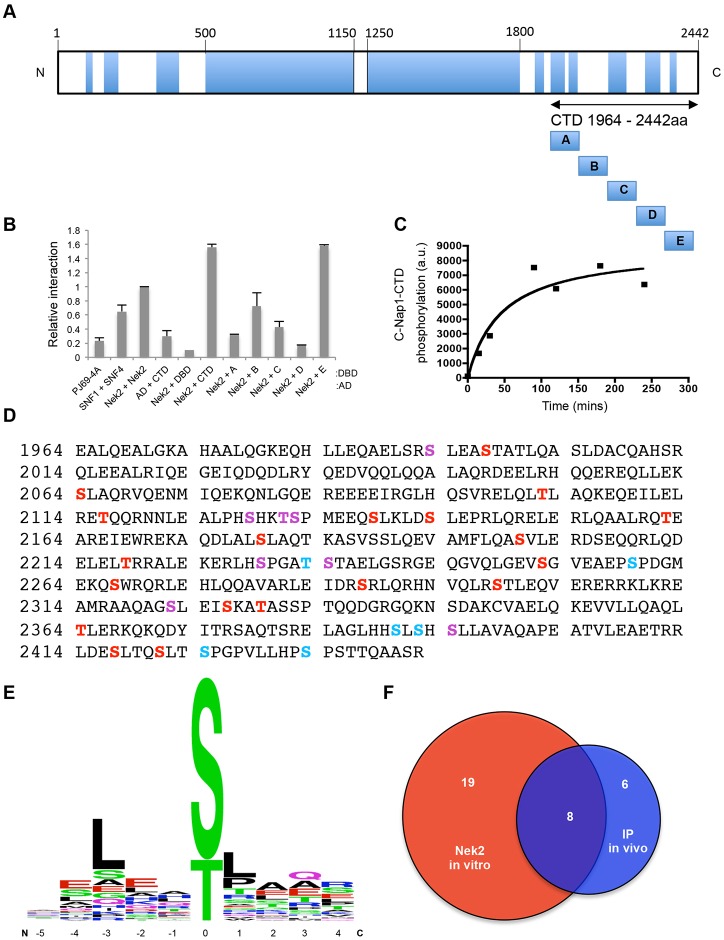
**Multisite phosphorylation of the C-Nap1 CTD by Nek2.** (A) Schematic diagram of C-Nap1 protein with predicted coiled-coils (blue). The C-terminal domain (CTD), residues 1964–2442, and five sub-fragments of this region are indicated: A (1964–2070), B (2062–2168), C (2162–2268), D (2262–2368) and E (2362–2442). (B) Yeast two-hybrid interaction activity between the pairs of constructs indicated is shown relative to the homodimeric interaction of Nek2A (arbitrarily assigned as 1). The first protein in each pair is a GAL4 activation domain (AD) fusion and the second protein is a GAL4 DNA-binding domain (DBD) fusion. Activity from untransformed yeast (PJ69-4A), the known interaction of SNF1 and SNF4, the CTD with AD alone, and Nek2 with the DBD alone are shown. Fragments A to E are those shown in panel A. Data show the mean±s.d. (three independent experiments). (C) Timecourse of *in vitro* phosphorylation of a purified GST-tagged C-Nap1 CTD protein by recombinant Nek2A. Graph indicates ^32^P incorporation in arbitrary units (a.u.), as determined by scintillation counting of bands excised from an SDS-polyacrylamide gel. (D) Amino acid sequence of the C-Nap1 CTD highlighting residues that are phosphorylated *in vivo* (blue), *in vitro* by Nek2 (red) and both (purple). (E) Weblogo analysis of the Nek2 consensus phosphorylation site, based on sites phosphorylated by Nek2 within the C-Nap1 CTD *in vitro*. (F) Venn diagram showing the relative proportion of phosphorylation sites found on the CTD phosphorylated by Nek2 *in vitro* and those identified *in vivo* (colors as in D).

We next sought to determine how C-Nap1 recruits Nek2 to the centrosome. C-Nap1 was identified through interaction of its CTD (residues 1964–2442) with Nek2 in a yeast two-hybrid assay (Fry et al., 1998a). Using this assay to more precisely map the region of interaction, we found that Nek2 primarily binds the C-terminal 80 residues of C-Nap1 (residues 2362–2442; fragment ‘E’ in Fig. 2A,B). However, expression of a Myc-tagged version of fragment E did not appear to act in a dominant-negative manner, as it did not displace endogenous Nek2 from the centrosome (supplementary material Fig. S1B).

We then investigated which site(s) within the entire CTD (residues 1964–2442) might be phosphorylated by Nek2 by performing *in vitro* kinase assays over 4 hours to achieve maximal phosphorylation (Fig. 2C). Analysis by mass spectrometry led to the identification of 27 sites of serine or threonine phosphorylation (Fig. 2D). As a result, this allowed us to assess the sequence preference for Nek2 phosphorylation on a physiological protein substrate. This indicated a strong, albeit not absolute, requirement for a leucine at −3, with 14 of the 27 sites falling into an LxxS/T consensus (Fig. 2E). There was also a strong preference for a hydrophobic residue at +1, with 16 out of 27 sites having a hydrophobic residue at this position. To determine whether some of these sites are phosphorylated *in vivo*, we subjected a Myc-tagged version of the C-Nap1 CTD to mass spectrometry following immunoprecipitation from transfected cells. This led to the identification of 14 sites of serine or threonine phosphorylation, four of which had the LxxS/TΦ consensus (Fig. 2D). There was considerable overlap between the sites identified *in vitro* and *in vivo*, with eight sites identified from the immunoprecipitated sample being phosphorylated by Nek2 *in vitro* (Fig. 2F). Furthermore, 15 sites of serine or threonine phosphorylation in this region of C-Nap1 have also been reported from global phosphoproteome studies and curated on the PhosphoSitePlus database (www.phosphosite.org). Of these, ten match sites that were phosphorylated by Nek2 *in vitro* and three others match sites that we identified *in vivo*. We conclude that Nek2 not only phosphorylates many sites within the CTD of C-Nap1, but that it is likely responsible for the bulk of phosphorylation on this domain *in vivo*. However, it remains possible that other kinases can also phosphorylate this domain in cells.

### Phosphorylation of C-Nap1-CTD regulates its affinity for the centrosome

**Fig. 3.**
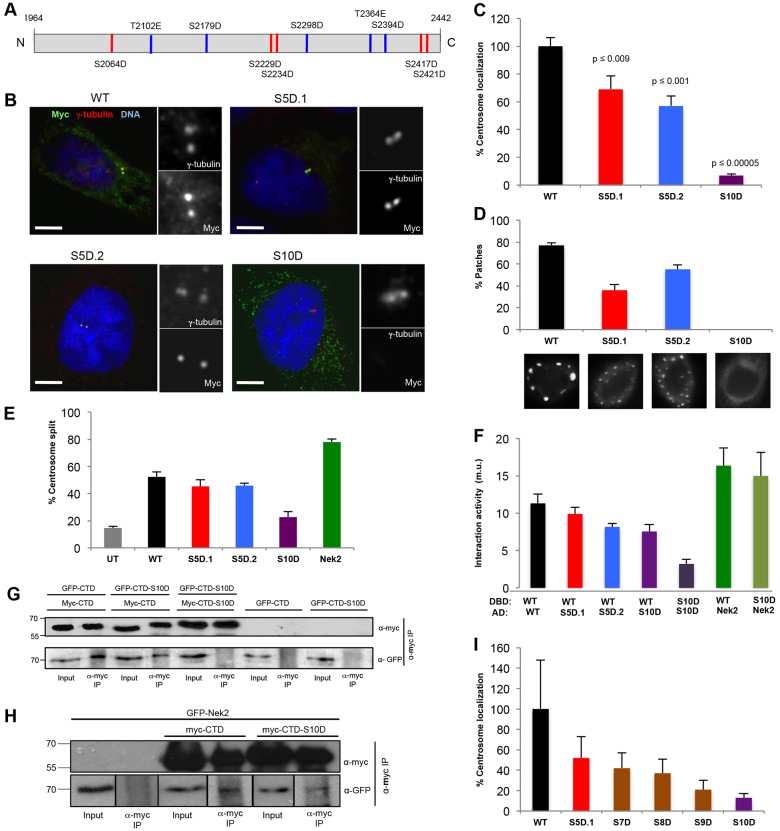
**Phosphomimetic mutations prevent C-Nap1-CTD oligomerization and centrosome localization.** (A) Schematic diagram showing the ten phosphorylation sites mutated in the Myc-tagged C-Nap1-CTD-S10D mutant. Red, sites mutated in the S5D.1 mutant; blue, sites mutated in the S5D.2 mutant. (B) U2OS cells were transfected for 24 hours with the Myc-tagged C-Nap1-CTD constructs indicated and stained with antibodies against γ-tubulin (red) and Myc (green), and with Hoechst 33342 to visualize DNA (blue). Magnified views of centrosomes are shown. Scale bars: 5 µm. (C) The percentage of transfected cells in which the recombinant protein was detected at the centrosome is shown (*n* = 50). *P*-values for each construct compared with the wild type (WT) are indicated (Student's *t*-test). (D) The percentage of transfected cells positive for large patches of recombinant protein, together with representative images of each construct stained with antibodies against Myc, is shown (*n* = 30). (E) The percentage of transfected cells in which the two centrosomes were split (>2 µm) is indicated (*n* = 100). UT, untransfected cells. Cells transfected with Myc–Nek2A were used as a positive control. (F) Yeast two-hybrid assay in L40 *S. cerevisiae* indicating interaction activity (m.u., Miller units) between the proteins shown where the upper protein was a LexA-DBD fusion and the lower a VP16-AD fusion. (G,H) GFP–C-Nap1-CTD (G) or GFP–Nek2A (H) were coexpressed with Myc–C-Nap1-CTD or CTD-S10D in U2OS cells for 24 hours, as indicated. Cell lysates were immunoprecipitated (IP) using antibodies against Myc, and inputs and immunoprecipitates were western blotted with anti-Myc and anti-GFP antibodies. Molecular mass (kDa) is indicated on the left. (I) The percentage of transfected cells in which the recombinant CTD protein was detected at the centrosome is shown (*n* = 50). Data in C–F and I show the mean±s.d. (at least three independent experiments).

Previous studies have indicated that the isolated C-Nap1 CTD, the secondary structure of which is mainly α-helical, can associate with centrosomes (Mayor et al., 2000). Also, when overexpressed, the CTD forms large oligomeric patches indicative of self-assembly, while triggering rootletin displacement and premature centrosome splitting (Mayor et al., 2002; Mayor et al., 2000; Yang et al., 2006). To examine how phosphorylation might regulate C-Nap1, a phosphomimetic mutant was generated in which 10 of the 27 serines and threonines phosphorylated by Nek2 *in vitro* and distributed across this domain were mutated to acidic residues (Fig. 3A). When expressed in HeLa cells, this construct, CTD-S10D, was diffusely distributed in the cytoplasm and failed to localize to the centrosome (Fig. 3B,C; supplementary material Fig. S2A). It also failed to assemble into patches when expressed at high levels and induced only a relatively low level of centrosome splitting (Fig. 3D,E). Indeed, cells expressing the CTD-S10D protein still retained rootletin at the centrosome, presumably recruited by endogenous C-Nap1 (supplementary material Fig. S2B). Yeast two-hybrid and co-immunoprecipitation assays confirmed that the S10D mutant had a reduced capacity for self-association. However, there was no loss of association of the S10D mutant with Nek2 in either yeast two-hybrid or co-immunoprecipitation experiments (Fig. 3F–H). This not only confirmed that this protein retains functionality, but indicated that the interaction site of Nek2 within the C-terminal 80 residues of C-Nap1 is not perturbed by the increased negative charge that results from these mutations.

To determine whether one or more specific sites might be crucial, we generated two further phosphomimetic mutants – CTD-S5D.1, which had five of the ten sites mutated in the S10D construct, and CTD-S5D.2, which had the other five sites mutated (Fig. 3A). We found that these constructs were expressed at comparable levels and gave similar results to each other with a level of centrosome association, patch formation and centrosome splitting that was only modestly reduced compared with that of the wild-type CTD (Fig. 3C–E; supplementary material Fig. S2A). Moreover, we observed a clear dose-dependent response in terms of loss of centrosome association as the number of phosphomimetic mutations increased from five to ten (Fig. 3I; supplementary material Fig. S2C). Thus, we conclude that the affinity of this domain for the centrosome is reduced in proportion to the number of sites phosphorylated.

Another kinase, SIK2, can phosphorylate C-Nap1 and has been reported to regulate its localization to the centrosome (Ahmed et al., 2010). SIK2 primarily phosphorylates S2392, although it also phosphorylates C-Nap1 at S2234 and S2394. All three of these sites were identified in our *in vivo* analysis but only two (S2234 and S2394) were identified as Nek2 phosphorylation sites *in vitro*. This is consistent with SIK2 being the primary kinase that phosphorylates S2392 in cells. To test the importance of the S2392 site alone, we generated a single phosphomimetic point mutant, CTD-S2392D. However, this mutant behaved in a similar manner to the wild-type protein, localizing strongly to the centrosome, assembling into patches and inducing centrosome splitting (supplementary material Fig. S2A; Fig. S3). Hence, phosphorylation at this site alone is insufficient to regulate C-Nap1. Nevertheless, SIK2 might cooperate with Nek2 to achieve a threshold of C-Nap1 phosphorylation necessary to trigger linker disassembly.

### Bulk charge regulates the affinity of the C-Nap1 CTD for the centrosome

**Fig. 4.**
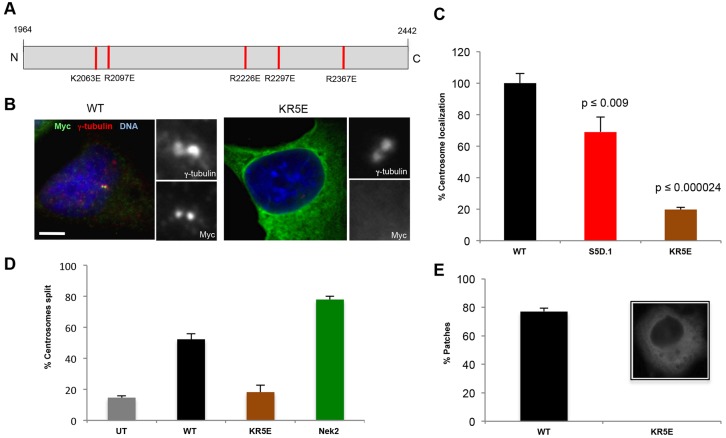
**Increased acidity is sufficient to prevent C-Nap1-CTD oligomerization and centrosome localization.** (A) Schematic diagram showing the five basic-to-acidic substitutions made in the KR5E mutant. (B) U2OS cells were transfected with the indicated constructs and stained using antibodies against γ-tubulin (red) and Myc (green), along with the DNA stain Hoechst 33342 (blue). Magnified views of centrosomes are shown. Scale bar: 5 µm. (C–E) Percentages of transfected cells exhibiting centrosome localization (C; *n* = 50), centrosome splitting (D; *n* = 100) and patch formation (E; *n* = 30) for the indicated constructs are shown, and were determined as described in Fig. 3C–E. A representative immunofluorescence image of a cell transfected with Myc–C-Nap1-CTD-KR5E and stained with anti-Myc antibodies highlighting the diffuse cytoplasmic stain is shown in panel E. WT, wild type; UT, untreated. Data show the mean±s.d. (at least three independent experiments).

Based on the data presented above, we reasoned that an increase in negative charge on the C-Nap1 CTD causes its displacement from the centrosome. To test this hypothesis, we mutated five lysines or arginines, which were not associated with particular phosphorylation sites and were distributed across the CTD, to glutamate (Fig. 4A). Strikingly, this construct, CTD-KR5E, behaved in a very similar manner to the CTD-S10D mutant that has an equivalent change in charge, localizing diffusely in the cytoplasm and failing to associate with the centrosome (Fig. 4B,C; supplementary material Fig. S2A). It also did not form patches and neither did it induce centrosome splitting (Fig. 4D,E). Hence, these data argue that there is unlikely to be a single key site of phosphorylation that regulates C-Nap1 localization. Rather, we propose that cumulative phosphorylation increases the overall negative charge until it reaches a threshold that prevents it from associating with a centrosomal partner.

### Phosphorylation of C-Nap1 displaces it from spindle poles

**Fig. 5.**
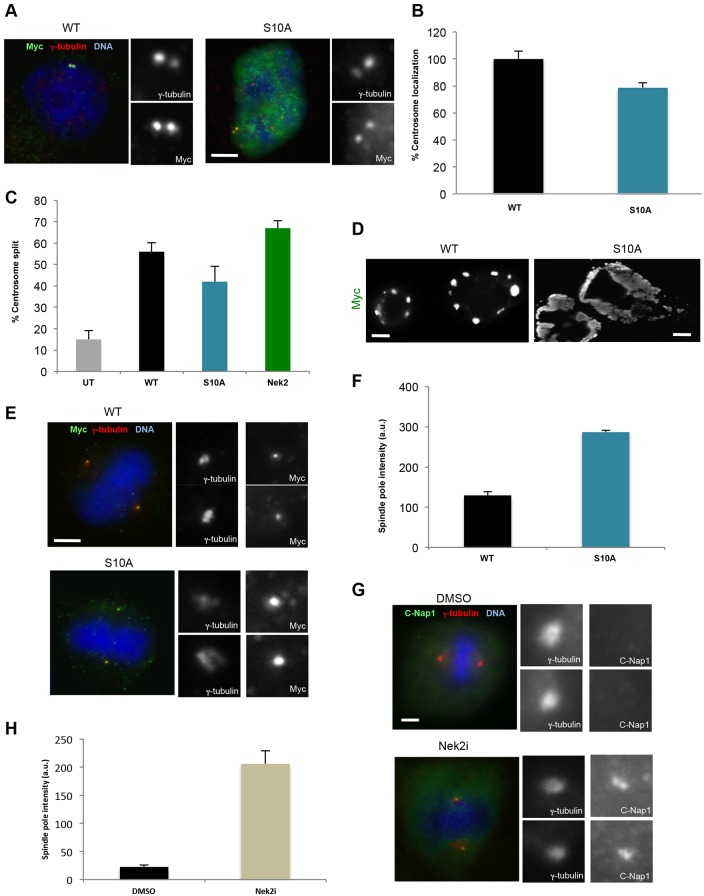
**Non-phosphorylatable C-Nap1-CTD is retained on spindle poles.** (A) U2OS cells were transfected with the constructs indicated and stained using antibodies against γ-tubulin (red) and Myc (green), along with the DNA stain Hoechst 33342 (blue). WT, wild type. (B) The percentage of transfected cells in which the recombinant protein was detected at the centrosome is shown (*n* = 50). (C) The percentage of interphase cells transfected with the constructs indicated in which the two centrosomes were split (>2 µm) is shown (*n* = 100). UT, untransfected cells. (D) U2OS cells were transfected with the constructs indicated and stained with anti-Myc antibodies (grayscale) revealing the extensive patch formation by the CTD-S10A protein. (E) U2OS cells were transfected with the indicated constructs and stained using antibodies against γ-tubulin (red) and Myc (green), along with the DNA stain Hoechst 33342 (blue). Metaphase cells are shown. (F) Mean spindle pole intensities (a.u., arbitrary units) of transfected proteins in metaphase cells are indicated, based on Myc staining (*n* = 20). (G) U2OS cells were synchronized in G2 with RO-3306 for 16 hours before release into fresh medium with 10 µM Nek2 inhibitor, R21, (or DMSO as control) for 3 hours. Cells were stained with antibodies against γ-tubulin (red) and C-Nap1 (green), and the DNA stain Hoechst 33342 (blue). Magnified views of centrosomes are shown in A, E and G. Scale bars: 5 µm. (H) Spindle pole intensity of endogenous C-Nap1 was determined in 20 metaphase cells treated as in G. All data show the mean±s.d. (at least three independent experiments).

To test whether the phosphorylation of C-Nap1 by Nek2 regulates its affinity for centrosomes in mitosis, we generated a CTD-S10A mutant in which the same ten phosphorylation sites that were previously mutated to acidic residues were converted to alanine. When expressed at low levels in interphase cells, this phospho-null mutant exhibited similar recruitment to the centrosome as the wild-type protein (Fig. 5A,B) and induced a similar level of split centrosomes (Fig. 5C). However, when expressed at higher levels, the protein assembled into large patches that occupied much of the cytoplasm (Fig. 5D). In transfected mitotic cells, the mutant CTD protein accumulated strongly on spindle poles to levels approximately threefold higher than those of the wild-type protein, although it did not prevent centrosome separation (Fig. 5E,F). Consistent with this, treatment of cells with a small molecule inhibitor of Nek2, aminopyridine (R)-21 (Innocenti et al., 2012), also led to greatly enhanced accumulation of endogenous C-Nap1 at spindle poles without blocking centrosome separation (Fig. 5G,H; supplementary material Fig. S4A). Taken together, these data support the hypothesis that phosphorylation of C-Nap1 by Nek2 displaces it from centrosomes during mitotic progression and, although not essential for centrosome separation, this might contribute to its timing.

### Phosphorylation of C-Nap1 increases towards mitosis

**Fig. 6.**
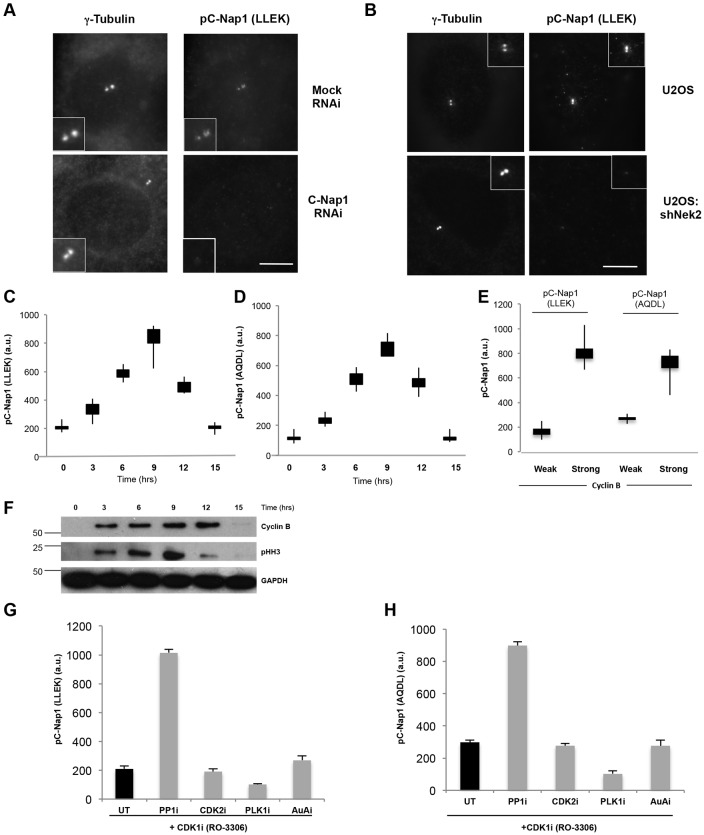
**Centrosomal phosphorylation of C-Nap1 increases in G2 cells.** (A) U2OS cells were transfected with siRNA oligonucleotides, as indicated, for 48 hours before being fixed and analyzed by immunofluorescence microscopy with antibodies against γ-tubulin and the anti-pC-Nap1 antibody LLEK. (B) Parental U2OS cells and a doxycycline-inducible Nek2-shRNA-expressing U2OS cell line in which Nek2 was knocked down for 48 hours were fixed and analyzed as in A. Magnified views of centrosomes are shown as insets. Scale bars: 5 µm. (C,D) U2OS cells were treated with aphidicolin for 16 hours, washed and released into fresh medium. Cells were collected at specified times, fixed and stained with the anti-pC-Nap1 antibodies LLEK (C) or AQDL (D). Centrosome intensities of the anti-pC-Nap1 antibodies in 100 cells were scored as indicated. The box and whisker plots represent the spread of centrosome intensities, where the box represents data from 25–75% and the whiskers show the minimum to maximum values. a.u., arbitrary units. (E) Box and whisker plots as in C,D, except showing pC-Nap1 centrosome intensity in cells released for 9 hours from an S phase arrest. Cells were co-stained with the anti-pC-Nap1 antibodies indicated and anti-cyclin-B antibodies, and scored as having strong or weak cyclin B staining. (F) Lysates were prepared from cells treated as in C,D and western blotted for cyclin B, phospho-histone H3 (pHH3) and GAPDH. Molecular mass (kDa) is indicated. (G,H) U2OS cells were treated with the Cdk1 inhibitor, RO-3306, for 16 hours to arrest cells in G2, and then inhibitors (i) against PP1 (calyculin A), Cdk2 (NU6120), PLK1 (BI 2536) or Aurora A (AuA; MLN 8054) were added for 30 minutes. Cells were stained with antibodies against γ-tubulin, to detect the centrosome, and anti-pC-Nap1 antibodies. The centrosome intensities of pC-Nap1 (LLEK, panel F; AQDL, panel G) are shown. UT, untreated. Data show the mean±s.d. (at least three independent experiments).

To assess centrosomal C-Nap1 phosphorylation through the cell cycle, we generated three phospho-specific antibodies against sites phosphorylated by Nek2. We have previously reported two of these antibodies. The ‘RRLD’ anti-phosphorylated (p)C-Nap1 antibody (directed against a peptide containing both S2417 and S2421) is specific for phosphorylated C-Nap1 in western blotting, and it confirmed the phosphorylation of C-Nap1 at this site in mitotic cells (Mardin et al., 2010). However, this antibody was not specific in immunofluorescence microscopy. By contrast, the ‘AQDL’ anti-pC-Nap1 antibody (directed against S2179) was specific for phosphorylated C-Nap1 in immunofluorescence microscopy, revealing centrosome staining in interphase cells that could be prevented by C-Nap1 and Nek2 depletion, or by addition of a Nek2 inhibitor (Innocenti et al., 2012). Here, we describe a third anti-pC-Nap1 antibody, ‘LLEK’, directed against S2064. As with the AQDL antibody, the LLEK antibody detects centrosomes in interphase cells by immunofluorescence microscopy, and this staining is abolished by depletion of C-Nap1 or Nek2 (Fig. 6A,B). Using the AQDL and LLEK antibodies as surrogate markers, we analyzed centrosomal C-Nap1 phosphorylation in synchronized cells. Both phospho-specific antibodies revealed a steady increase in centrosome intensity as cells were released from an S phase arrest and progressed towards mitosis (0–9 hours). However, pC-Nap1 staining at the centrosome decreased at later time-points (12–15 hours) when cells pass through mitosis into G1 (Fig. 6C,D). At the 9-hour time-point, co-staining with cyclin B revealed fourfold higher centrosomal pC-Nap1 in cells with strong cyclin B staining, indicative of cells in late G2, as compared with cells with weak cyclin B, indicative of either S phase or G1 (Fig. 6E). Western blotting for cyclin B and phospho-histone-3 (pH3) confirmed that cells progressed through mitosis at the 9–12-hour time-points post-release (Fig. 6F). Taken together, these data show that centrosomal C-Nap1 phosphorylation is cell cycle dependent with an increase in S and G2 phases, before it decreases in late mitosis and G1.

To determine whether the extent of C-Nap1 phosphorylation might be subject to additional regulation, we treated G2-arrested cells with the PP1 inhibitor calyculin A. This led to a fourfold to fivefold increase in centrosomal pC-Nap1 staining with both antibodies (Fig. 6G,H). Inhibitors of Cdk2 or Aurora A had no effect on centrosomal pC-Nap1, despite having activity in other assays performed in our laboratory (Prosser et al., 2012). Interestingly, the centrosomal intensity of pC-Nap1 was reduced upon inhibition of Plk1, consistent with Plk1 contributing to the centrosomal recruitment of Nek2 (Mardin et al., 2011). Hence, we propose that C-Nap1 phosphorylation increases as cells progress towards mitosis, but that inactivation of PP1 is required to overcome the threshold of phosphorylation necessary for centrosome disjunction at the onset of mitosis.

### Phosphorylation of C-Nap1 regulates its affinity for Cep135

**Fig. 7.**
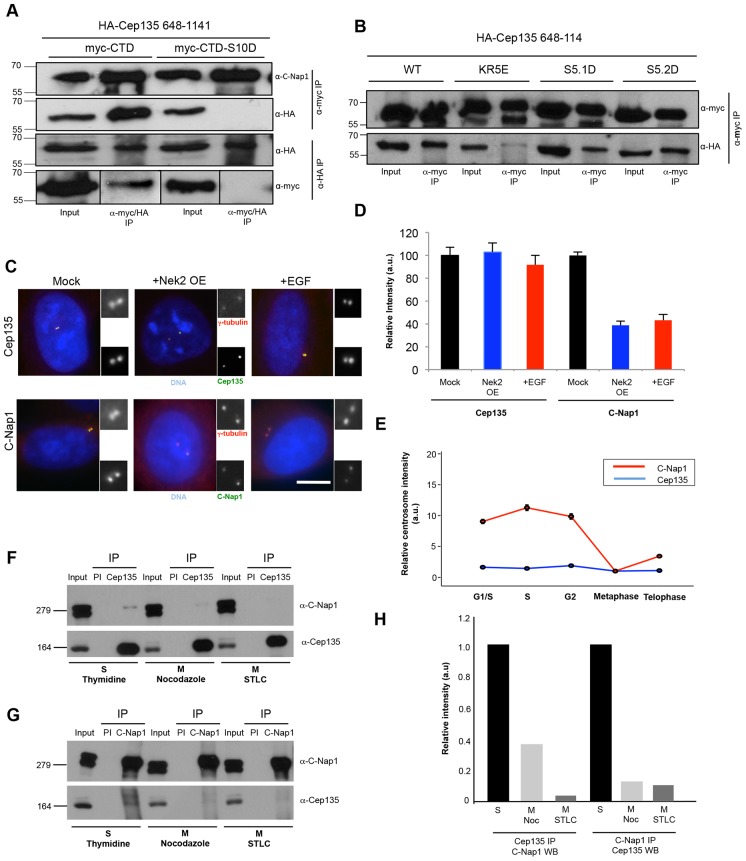
**C-Nap1 phosphorylation disturbs its interaction with Cep135 in mitosis.** (A) HA–Cep135-CTD (648–1141) was co-transfected with Myc–C-Nap1-CTD or CTD-S10D into U2OS cells. Cell lysates were immunoprecipitated (IP) using antibodies against Myc or HA, and western blotted with antibodies against C-Nap1, Myc or HA, as indicated. Note that in the anti-Myc blot, shorter exposures are shown for the inputs, but exposures for the two IPs are the same. (B) As for A, except using the Myc–C-Nap1-CTD constructs indicated and immunoprecipitations were only performed with anti-Myc antibodies. WT, wild type. (C) U2OS cells were mock treated, transfected for 24 hours to overexpress Nek2 (Nek2 OE) or stimulated with EGF for 4 hours before being fixed and stained with antibodies against γ-tubulin (red) and Cep135 or C-Nap1 (green), along with the DNA stain Hoechst 33342 (blue). Scale bar: 5 µm. (D) Centrosome intensities of Cep135 and C-Nap1 in the cells overexpressing Nek2 or treated with EGF were determined relative to the mock-treated cells. a.u., arbitrary units. Data show the mean±s.d. (three independent experiments). (E) HeLa cells were synchronized with a double-thymidine block and released for 0 hours (G1/S), 4 hours (S) or 9 hours (G2). Metaphase and telophase cells were identified in an unsynchronized population by chromosome status. In each case, cells were fixed and stained with Cep135 and C-Nap1 antibodies, and cell cycle status was confirmed by staining for cyclin B1. Centrosome intensities were measured and are presented as means relative to that of metaphase cells (*n* = 50). (F,G) HEK 293T cells that were arrested in the cell cycle as indicated were lysed and immunoprecipitated using pre-immune serum (PI) and anti-Cep135 (F) or anti-C-Nap1 (G) antibodies. Lysates (input) and immunoprecipitates were western blotted with antibodies against Cep135 and C-Nap1, as indicated. Molecular mass (kDa) is shown on the left of panels A, B, F and G. (H) Quantification of data from F and G showing the extent of co-precipitation in mitotically-arrested cells relative to that seen in S-phase-arrested cells. Noc, nocodazole; WB, western blotted.

To understand the mechanism by which C-Nap1 phosphorylation regulates its displacement from the centrosome, we examined its interaction with Cep135, a proximal-end centriole marker that has been reported to associate with C-Nap1 (Kim et al., 2008). Structure-function studies had indicated that the CTD of C-Nap1 interacts with the C-terminal region (residues 648–1141) of Cep135. Using this Cep135 C-terminal construct, we therefore tested its ability to co-precipitate with the wild-type C-Nap1 CTD and the different CTD mutants. Strikingly, we found that although the wild-type C-Nap1 CTD bound to Cep135, both the S10D and KR5E mutants lost interaction, whereas the S5D.1 and S5D.2 mutants exhibited reduced interaction (Fig. 7A,B; supplementary material Fig. S4B). We then examined the localization of C-Nap1 and Cep135 in cells in which centrosomes were prematurely split in response to Nek2 overexpression or addition of epidermal growth factor (EGF), both of which induce centrosome splitting in a Nek2-dependent manner (Faragher and Fry, 2003; Mardin et al., 2013). In response to these treatments, we found that although C-Nap1 was displaced from the split centrosomes, the levels of Cep135 were unchanged (Fig. 7C,D). Moreover, direct comparison of the centrosomal intensities of these two proteins in synchronized cells confirmed that C-Nap1 was lost from centrosomes as they passed from G2 into M, whereas Cep135 was retained (Fig. 7E; supplementary material Fig. S4C). Importantly, by performing reciprocal co-immunoprecipitation experiments, we found that endogenous C-Nap1 and Cep135 associated in S-phase-arrested cells, but not in cells arrested in M phase with nocodazole or the Eg5 inhibitor *S*-trityl-l-cysteine (STLC) (Fig. 7F–H). Interestingly, when immunoprecipitation was performed with anti-C-Nap1 antibodies, the co-precipitated population of Cep135 exhibited a partial gel-mobility shift. This raises the possibility that C-Nap1 preferentially binds to a modified fraction of Cep135 that is found only at the centrosome in interphase cells. Thus, we conclude that centrosome disjunction is triggered by loss of C-Nap1 interaction with Cep135 that results from Nek2-induced hyperphosphorylation of C-Nap1.

## DISCUSSION

**Fig. 8.**
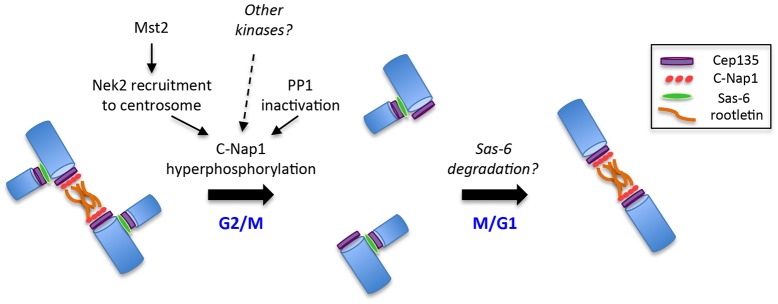
**Induction of centrosome disjunction by C-Nap1 hyperphosphorylation.** The left-hand side of this schematic model summarizes our current understanding that Mst2-driven recruitment of Nek2, together with inactivation of PP1 and potentially phosphorylation by other kinases, leads to C-Nap1 hyperphosphorylation at the G2/M transition. This disturbs the interaction of C-Nap1 with Cep135, with C-Nap1 itself and possibly with rootletin, leading to disassembly of the intercentriolar linker and driving centrosome disjunction. On the right-hand side, we hypothesize that Sas-6 and C-Nap1 might compete for binding to Cep135, and that degradation of Sas-6 at the end of mitosis would enable C-Nap1 recruitment and re-establishment of the linker as cells pass from mitosis back into G1. Blue, centrioles.

In this study, we provide mechanistic insight into the process of centrosome disjunction that breaks the proteinaceous linker that holds centrosomes together during interphase (Bruinsma et al., 2012; Mardin and Schiebel, 2012). Our data support a model in which centrosome disjunction is triggered by the hyperphosphorylation of C-Nap1, a major linker component. This occurs in response to a shift in the balance of activities of the Nek2–PP1 bi-stable switch. C-Nap1 hyperphosphorylation triggers the loss of both oligomerization and, crucially, interaction with the core centriole proximal-end protein, Cep135. Upon loss of this interaction, and potentially upon loss of oligomerization and association with rootletin, the integrity of the centriolar linker is broken and centrosomes are free to separate and establish the opposing poles of the mitotic spindle (Fig. 8).

Our results firstly demonstrate that the large coiled-coil proteins C-Nap1 and rootletin act as stable components of the intercentriolar linker. They also provide further evidence that although C-Nap1 is focused at the proximal ends of centrioles (Fry et al., 1998a; Mayor et al., 2000), rootletin forms filaments that can extend between the two centrosomes (Bahe et al., 2005; Yang et al., 2006). These linker components are, in turn, responsible for recruiting the protein kinase Nek2 (our data; Spalluto et al., 2012). Nek2 specifically interacts with the C-terminal 80 amino acids of C-Nap1, enabling it to regulate linker integrity through phosphorylation of C-Nap1 and rootletin. Furthermore, as the centrosome recruitment of Nek2 is stimulated through phosphorylation of its C-terminal non-catalytic domain by Mst2 (Hames et al., 2005; Mardin et al., 2010), Mst2 might directly regulate the affinity of Nek2 for C-Nap1.

Nek2 can phosphorylate many sites (>25) within the ∼50-kDa C-Nap1 CTD. This notion of multisite phosphorylation confirms earlier studies that suggest that Nek2 catalyzes the incorporation of at least 13 moles of phosphate per mole of this domain (Helps et al., 2000). Importantly, many of the same sites were found to be phosphorylated in cells either by us or in global phosphoproteome studies. The lack of structural information means that we cannot tell how these sites are distributed across the domain surface. However, we found that different sets of phospho-site mutants, as well as a construct that had an equivalent charge change due to basic-to-acidic substitutions had similar consequences on C-Nap1 localization and interactions. These data strongly suggest that Nek2 does not regulate C-Nap1 through site-specific phosphorylation, but rather through a local increase in negative charge. One consequence of this is to prevent oligomerization, presumably through electrostatic repulsion. Self-assembly of C-Nap1, as well as association with the closely related linker component rootletin is likely to be a major contributing factor to integrity of the linker. It is unclear though how C-Nap1 and rootletin interact. One group found strong interaction between multiple domains of C-Nap1 – including the CTD – with rootletin, whereas another group reported preferential interaction of rootletin with an N-terminal domain, but not the CTD, of C-Nap1 (Bahe et al., 2005; Yang et al., 2006). Meanwhile, centlein interacts with the C-Nap1 CTD and acts as a bridge to Cep68 (Fang et al., 2014). Hence, we hypothesize that Nek2-mediated phosphorylation might perturb the interaction of C-Nap1 with rootletin and centlein, while these other linker proteins might equally be subject to (multi)-site phosphorylation by Nek2. Either of these events would further promote linker disassembly and centrosome disjunction.

Identification of a large number of phosphorylation sites allowed us to analyze the preferred sequence for Nek2 phosphorylation based on a protein substrate rather than peptide libraries. This led to the definition of a consensus motif of LxxS/TΦ. An oriented peptide-library screen identified [F/L/M]xxS/TΦ[R/H/X] as the optimal motif for Nek2, which fits very well with the consensus identified from our studies (Alexander et al., 2011). However, what was clear from our analysis was the strong preference for leucine at position −3, with none of the 27 sites having F or M at this position. In fact, 13 of the 27 sites had none of these three residues at this position. Also, the peptide screen found a strong discrimination against proline at position +1, and yet two of the sites we identified had a proline at this position. Hence, peptide screens provide useful information on consensus sequences, but additional factors can influence phospho-site selection in intact proteins.

The generation of phospho-specific antibodies allowed us to address the extent of C-Nap1 phosphorylation at centrosomes in cells. This revealed a number of interesting points about the regulation of centrosome cohesion. Firstly, phosphorylation of centrosomal C-Nap1 was detected in interphase before centrosome disjunction occurred. Secondly, the extent of phosphorylation increased as cells progressed from S to G2. Thirdly, incubation of cells with a PP1 inhibitor led to a further substantial increase in centrosomal C-Nap1 phosphorylation. We propose that a low level of C-Nap1 phosphorylation is present from S phase onwards, when Nek2 is first expressed and recruited to the centrosome. The steady increase in this phosphorylation could reflect increased centrosome recruitment of Nek2 in response to phosphorylation by Mst2. However, during interphase, the overall stoichiometry of phosphorylation is below that required to trigger centrosome disjunction, most likely as a result of the counteracting phosphatase PP1. Only upon inhibition and/or removal of PP1 at mitosis does the phosphorylation of C-Nap1 overcome the threshold required to precipitate centrosome disjunction. Indeed, PP1 is both inhibited at this time through Cdk1-mediated recruitment of inhibitor-2, and released from the Nek2–Mst2 complex by Plk1-mediated phosphorylation of Mst2 (Eto et al., 2002; Mardin et al., 2011). Consistent with this, we found that C-Nap1 phosphorylation was reduced upon Plk1 inhibition. These data explain the somewhat surprising observation that the intrinsic catalytic activity of Nek2 remains constant through S and G2 and does not peak at mitotic entry (Fry et al., 1995).

A number of other sites were identified from the *in vivo* analysis that were not phosphorylated by Nek2 *in vitro*, including S2392 that was reported to be phosphorylated by the SIK2 kinase (Ahmed et al., 2010). We found that mutation of this site alone had little or no effect on centrosome localization or oligomerization. However, our model is consistent with the notion that additional kinases phosphorylating different sites can cooperate with Nek2 to help achieve the threshold of negative charge required for linker disassembly.

As well as stimulating loss of oligomerization, C-Nap1-CTD phosphorylation led to its displacement from the centrosome. It was reported that Cep135, a core centriolar protein present at the proximal ends, acts as a platform for the recruitment of C-Nap1 (Kim et al., 2008). Our data support and extend this hypothesis, revealing that phosphorylation of the C-Nap1 CTD specifically disrupts interaction with the C-terminal domain of Cep135. Again, our data with the KR5E mutant suggests that this is due to electrostatic repulsion, consistent with the acidic nature of the Cep135 CTD (pI of 5.72). Moreover, we show that endogenous C-Nap1 and Cep135 interact in asynchronous cells but not mitotically arrested cells. We therefore propose that a key mechanism in centrosome disjunction is the loss of interaction between C-Nap1 and Cep135 that results from Nek2-induced phosphorylation of C-Nap1. Intriguingly, the same C-terminal region of Cep135 also interacts with the Sas-6 protein (Lin et al., 2013). Sas-6 localizes to the proximal ends of nascent procentrioles during the process of centriole duplication but is not present on parental centrioles. Hence, these two proteins might interact with Cep135 at different times in the centriole cycle and, potentially, in a mutually exclusive manner. We are currently exploring the hypothesis that degradation of Sas-6, which occurs in late mitosis concomitant with centriole disengagement (Strnad et al., 2007), allows the recruitment of C-Nap1 and re-establishment of the linker at the start of the next cell cycle.

Accurate segregation of chromosomes requires timely centrosome separation (Indjeian and Murray, 2007; Kaseda et al., 2012; Mardin et al., 2013; Silkworth et al., 2012). Centrosomes can separate early in prophase, prior to nuclear envelope breakdown, or during prometaphase, once microtubules have access to the chromosomes (Tanenbaum and Medema, 2010). Although the events required for separation of centrosomes in prophase or prometaphase might differ, they both depend on the action of microtubule-based motors, such as Eg5 and hKlp2 (also known as KIF15), to drive centrosomes apart through anti-parallel microtubule sliding. These motors are capable of forcing bipolar spindle assembly even in cells with amplified centrosomes through a mechanism of centrosome clustering (Ganem et al., 2009). Moreover, the concerted action of microtubules and motors can promote full centrosome separation in the absence of Nek2, explaining why Nek2 depletion does not block mitotic progression. One can speculate that the separating forces provided by these motors are sufficient to tear the linker apart when its timely disassembly has not occurred. However, when motor activity is reduced (e.g. in the presence of low-dose kinesin inhibitors), disassembly of the linker and Nek2 activity become essential for bipolar spindle assembly and chromosome stability (Mardin et al., 2013; Mardin et al., 2010). Equally, elevated expression of Nek2 might lead to premature centrosome separation that could affect cell polarity, migration and cell division. Thus, the overexpression of Nek2 that is frequently observed in human cancers could make a significant contribution to tumor progression (Hayward and Fry, 2006; Zhou et al., 2013).

## MATERIALS AND METHODS

### Plasmid construction and mutagenesis

The C-Nap1 CTD (residues 1964–2442) was subcloned into pLeics 20 or pGEX for expression in mammalian cells with an N-terminal Myc tag or bacteria with an N-terminal GST tag, respectively. The rootletin CTD (residues 1651–2018) was fused to an N-terminal maltose-binding protein (MBP) by subcloning into the pMALc2X vector, expressed in bacteria and purified by amylose resin affinity chromatography. Cep135 constructs have been described previously (Kim et al., 2008). Site-directed mutagenesis was performed using the Genetailor™ Site-Directed Mutagenesis Kit (Invitrogen) according to the manufacturer's instructions. All constructs were verified by DNA sequencing, and bacterial expression and purification were performed according to standard procedures.

### Cell culture and transfections

HeLa, U2OS and HEK 293T cells were grown in Dulbecco's modified Eagle's medium (DMEM) supplemented with 10% fetal calf serum, 100 IU/ml penicillin and 100 µg/ml streptomycin. Cells were grown at 37°C under 5% CO_2_. Transient transfections were performed with Fugene HD (Roche) according to the manufacturer's instructions, and cells were analyzed after 24 hours. For G1/S and G2 arrest, cells were incubated with 1.6 µg/ml aphidicolin (Sigma) and 10 µM RO-3306 (Calbiochem) for 16 hours, respectively. Specific inhibitors were used as indicated for Plk1 (100 nM BI 2536; Axon Med Chem), aurora A (1 µM MLN 8054; Prosser et al., 2012), Cdk2 (50 µM NU6120; Hardcastle et al., 2004), Nek2 (10 µM (R)-21; Innocenti et al., 2012) and PP1 (10 nM calyculin A; Meraldi and Nigg, 2001). Microtubules were depolymerized with nocodazole as described previously (Hames et al., 2005).

### RNAi

siRNA oligonucleotides against GAPDH (Ambion, AM4631), C-Nap1 (5′-GGAAGAGCGUCUAACUGAU-3′; Qiagen) and rootletin (5′-CAGCCAGGAGAAGAUCAGCAAUU-3′; 5′-CAGGGAGAUUGUCACCCGCAAUU-3′; Dharmacon) were transfected using Oligofectamine (Invitrogen).

### Cell lysis, immunoprecipitation and western blotting

Cell lysates were prepared as indicated in NEB buffer (Zalli et al., 2012), single-detergent lysis buffer (50 mM Tris-HCl pH 8.0, 150 mM NaCl, 0.02% sodium azide, 1% Triton X-100) or RIPA buffer (50 mM Tris-HCl pH 7.5, 150 mM NaCl, 1 mM EDTA, 0.1% SDS, 1% Triton X-100, 0.5% sodium deoxycholate) with protease inhibitors. Protein concentrations were determined by using the bicinchoninic acid (BCA) assay, and lysates were analyzed by SDS-PAGE and western blotting. For immunoprecipitation, lysates were pre-cleared for 30 minutes at 4°C with Protein-G–agarose (Sigma) or Protein-A–Sepharose (GE Healthcare) that had been pre-washed in lysis buffer. Beads were then removed and supernatants were incubated with antibodies against Myc (1∶1000; Cell Signaling Technology, 2276), HA (1∶1000; Cell Signaling Technology, 2362) or Cep135 (1∶1000; Kim et al., 2008) for 1 hour or overnight on ice. Immune complexes were then captured with pre-washed Protein A or Protein G beads for 1 hour at 4°C. Beads were washed four times with lysis buffer before analysis by SDS-PAGE and western blotting. For western blotting, the primary antibodies used were against Myc (1∶1000; Cell Signaling Technology, 2276), HA (1∶1000; Cell Signaling Technology, 3274), GFP (0.5 µg/ml; Abcam, 6556), cyclin B (10 µg/ml; Santa Cruz, SC245), phospho-histone 3 (10 µg/ml; Millipore, 06570), GAPDH (1∶1000; Cell Signaling Technology, 14C10), C-Nap1 (0.75 µg/ml; Fry et al., 1998a) and Cep135 (1∶500; Kim et al., 2008). Blots were developed using enhanced chemiluminescence (ECL) and autoradiography.

### Kinase assays and phospho-site mapping

Kinase assays were performed as described previously (Zalli et al., 2012), using 0.1 µg of Nek2 purified kinase (Cell Signaling, 7338). Briefly, proteins were incubated with 5 µg of C-Nap1 CTD and 1 µCi of [γ-^32^P]-ATP in 40 µl of kinase buffer (50 mM HEPES-KOH pH 7.4, 5 mM MnCl_2_, 5 mM β-glycerophosphate, 5 mM NaF, 4 µM ATP, 1 mM DTT) for 30 minutes at 30°C. Reactions were stopped with 50 µl of 3× sample buffer and analyzed by SDS-PAGE and autoradiography. Substrate phosphorylation was quantified by scintillation counting of protein bands excised from dried gels. Excised bands were immersed in 3 ml of Optiphase HiSafe 2 liquid scintillant (Wallace-Perkin Elmer) and ^32^P incorporation was determined by quantification in a LS6500 scintillation analyzer (Beckman Coulter). For phospho-site mapping, proteins were separated by SDS-PAGE, gels were stained with Coomassie Blue for 30 minutes and then destained. The C-Nap1 CTD protein was excised and phospho-sites were identified by liquid chromatography-tandem mass spectrometry (LC-MS/MS) analysis.

### Antibody production

Rootletin antibodies were generated against the MBP-tagged rootletin C-terminal fragment expressed in bacteria. Immunizations were performed in rabbits by Cambridge Research Biochemicals and antisera were purified on an affinity column made from the same C-terminal fragment of rootletin tagged with GST. The production and purification of antibody against pC-Nap1 was performed as described previously (Innocenti et al., 2012; Mardin et al., 2010), with the LLEK antibody raised against a phospho-peptide corresponding to residues LLEKpSLAQRVQC.

### Immunofluorescence microscopy

HeLa or U2OS cells were fixed with ice-cold methanol for 30 minutes at −20°C, and immunofluorescence microscopy was performed as described previously (O'Regan and Fry, 2009). Primary antibodies were against rootletin (1∶200, this study; or 1∶100, Santa Cruz, sc-67824), C-Nap1 (1∶1000, Fry et al., 1998a), Nek2 (1∶200, BD Transduction Labs, 610594), GFP (0.5 µg/ml, Abcam, 6556), Cep135 (1∶1000, Kim et al., 2008), Myc (1∶1000, Cell Signaling Technology, 2276), γ-tubulin (1∶1000, Sigma T6557), cyclin B1 (0.2 µg/ml, Abcam, 2949) and pC-Nap1 AQDL and LLEK (0.5 µg/ml, this study). Secondary antibodies used were Alexa-Fluor-488-conjugated and Alexa-Fluor-594-conjugated goat anti-mouse-IgG and goat anti-rabbit-IgG (10 µg/ml, Invitrogen A11001 and A11012, respectively) or Alexa-Fluor-488-conjugated donkey anti-goat-IgG and Rhodamine-conjugated donkey anti-rabbit-IgG (5 µg/ml, Jackson 705-545-003 and 711-025-152, respectively). Images were captured using Volocity software (Improvision) on an inverted Nikon TE300 or Olympus BX51 microscope with a 100× oil objective. Intensity measurements were quantified using Volocity or ImageJ (v1.4.1) software and images were processed in Adobe Photoshop 4.0.

### Photobleaching analysis

FRAP analysis was performed on a Leica SP5 confocal laser scanning microscope using a 100× oil objective (NA 1.4) and a scan zoom of 3. A square region of interest (ROI) of 50×50 pixels centered on both centrosomes was bleached with 40 iterations and 100% laser power (488-nm argon laser). Two images were captured before bleaching with a 1-second interval. An image was taken every second (488-nm argon laser at 4% power) after bleaching over a 30–90-second period. For each time-point, the fluorescence intensity of the photobleached ROI (P1) was determined using Leica software. The fluorescence intensity of an unbleached ROI in the cell was determined (U2) and a normalized intensity (In) was calculated using P1/U2. The background fluorescence intensity (Bk) was set as the first frame after photobleaching, and this value was subtracted from all frames, such that the fluorescence intensity for the ROI at a given time is equal to In−Bk. The amount of fluorescence recovery was then calculated as the fluorescence intensity value of a given frame divided by the fluorescence intensity value of the frame immediately before photobleaching and was expressed as a percentage (Thompson et al., 2004). Images were processed with Adobe Photoshop 4.0. *P*-values were calculated for *t*_1/2_ or percentage recovery where indicated using Student's *t*-test.

### Yeast two-hybrid analysis

Yeast two-hybrid interaction assays were performed using constructs generated in either pGBDU (GAL4-DBD) and pACTII (GAL4-AD) plasmids transformed into *Saccharomyces cerevisiae*, PJ69-4A, or in pLEICS-81 (LexA-DBD) and pLEICS-82 (VP16-AD) plasmids transformed into *S. cerevisiae*, L40. Interacting clones were selected by growth on synthetic dropout medium lacking appropriate amino acids, and interaction activity (Miller units) was measured using β-galactosidase assays.

## Supplementary Material

Supplementary Material
