# Supplementary material for: Multisite phosphorylation of C-Nap1 releases it from Cep135 to trigger centrosome disjunction
Source: J Cell Sci. 2014 Jun 1;127(11):2493–506. doi: 10.1242/jcs.142331 (PMC4038944; doi:10.1242/jcs.142331)
Supplement: Supplementary Material [file supp_127_11_2493__index.html]

Multisite phosphorylation of C-Nap1 releases it from Cep135 to trigger centrosome disjunction — Supplementary Material 

# Multisite phosphorylation of C-Nap1 releases it from Cep135 to trigger centrosome disjunction

## JCS142331 Supplementary Material

**Files in this Data Supplement:**

- **Supplementary Material**
