# Supplementary material for: Multisite phosphorylation of C-Nap1 releases it from Cep135 to trigger centrosome disjunction
Source: J Cell Sci. 2014 Jun 1;127(11):2493–506. doi: 10.1242/jcs.142331 (PMC4038944; doi:10.1242/jcs.142331)
Supplement: Supplementary Material [file supp_127.11.2493_JCS142331.pdf]

SUPPLEMENTARY INFORMATION

Multisite phosphorylation of C-Nap1 releases it from Cep135 to trigger centrosome disjunction

Tara Hardy[1], Miseon Lee[2], Rebecca S. Hames[1], Suzanna L. Prosser[1], Donna-Marie Cheary[1], Mugdha D. Samant[1], Francisca Schultz[1], Joanne E. Baxter[1], Kunsoo Rhee[2] and Andrew M. Fry[1]

Supplementary Figure Legends

Figure S1. Confirmation of microtubule depolymerization and analysis of cells expressing the C-terminal 80 residues of C-Nap1

A. U2OS cells were either untreated (panel *a*) or treated with nocodazole (panel *b*) to depolymerize microtubules at 1.6 µg/ml for 1 hour before fixation and staining with antibodies against α-tubulin (green) and γ-tubulin (red). **B**. U2OS cells were transfected with myc-C-Nap1-Fragment E (2362-2442) and stained with antibodies against myc (green) and either Nek2 or γ-tubulin (red). DNA was stained with Hoechst 33342. Scale bars, 10 µm.

Figure S2. Expression of C-Nap1-CTD mutants and consequences on rootletin localization

A. U2OS cells were transfected with the myc-tagged C-Nap1-CTD constructs indicated, lysed after 24 hours and the lysates analysed by Western blot with myc and GAPDH antibodies. M. wts (kDa) are indicated on the left. **B & C**. U2OS cells were transfected with the indicated myc-tagged C-Nap1-CTD constructs and stained using antibodies to myc (red) and rootletin (green in B) or γ-tubulin (green in C) along with the DNA stain, Hoechst 33342 (blue). Scale bars, 5 µm.

Figure S3. Phosphomimetic mutation of S2392 does not disturb C-Nap1-CTD

A. U2OS cells were transfected with the indicated myc-tagged C-Nap1-CTD constructs and stained using antibodies to γ-tubulin (red) and myc (green) along with the DNA stain, Hoechst 33342 (blue). Magnified views of centrosomes are shown. Scale bars, 5 µm. **B**. The percentage of transfected cells in which the recombinant CTD protein was detected at the centrosome is shown (n=50). **C**. The percentage of

transfected cells positive for large patches of recombinant CTD protein is given (n=30). A representative IF image of cells transfected with myc-C-Nap1-CTD-S2392 and stained with myc antibodies is shown. **D**. The percentage of transfected cells in which the two centrosomes were split (>2 µm) is indicated (n=100). UT, untransfected cells; cells transfected with myc-Nek2A were used as a positive control. Error bars in B-D are s.d. from at least 3 independent experiments.

Figure S4. Cell cycle-dependent phosphorylation of C-Nap1 regulates its centrosome localization and association with Cep135

A. U2OS cells were treated with either DMSO alone (panels *a* and *c*) or the Nek2 inhibitor (panels *b* and *d*) for 3 hours before staining with antibodies against γ-tubulin (red) and pC-Nap1 (green) along with the DNA stain, Hoechst 33342 (blue). Panels *a* and *b* are stained with the AQDL pC-Nap1 antibody, and panels *c* and *d* with the LLEK pC-Nap1 antibody. Scale bars, 5 µm. **B**. The histogram shows the amount of the HA-Cep135-CTD protein co-precipitated with the myc-C-Nap1-CTD constructs indicated, relative to the input detected for each co-transfection. These data relate to the co-immunoprecipitations shown in Fig. 7A & B of the main text. **C**. HeLa cells were synchronized with double thymidine block and released for 0 (G1/S), 4 (S) or 9 (G2) hours. Metaphase and telophase cells were identified in asynchronous populations. Cells were fixed and stained with CEP135 and C-Nap1 antibodies, while cell cycle stage was confirmed by cyclin B1 staining. DNA was stained with DAPI. Scale bars, 10 µm.

A

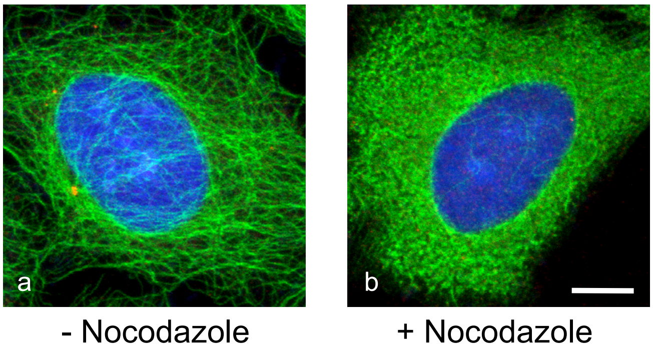
a
b
- Nocodazole
+ Nocodazole

B

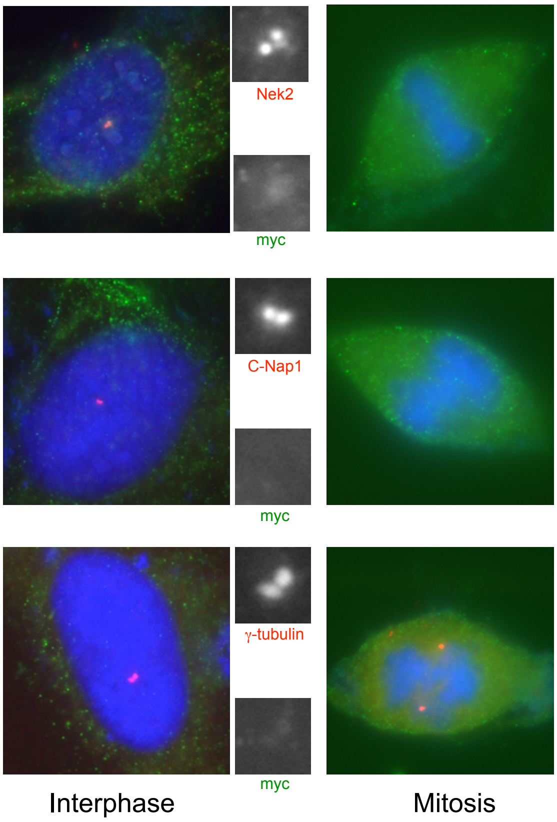
Nek2
myc
C-Nap1
myc
γ-tubulin
myc
Interphase
Mitosis

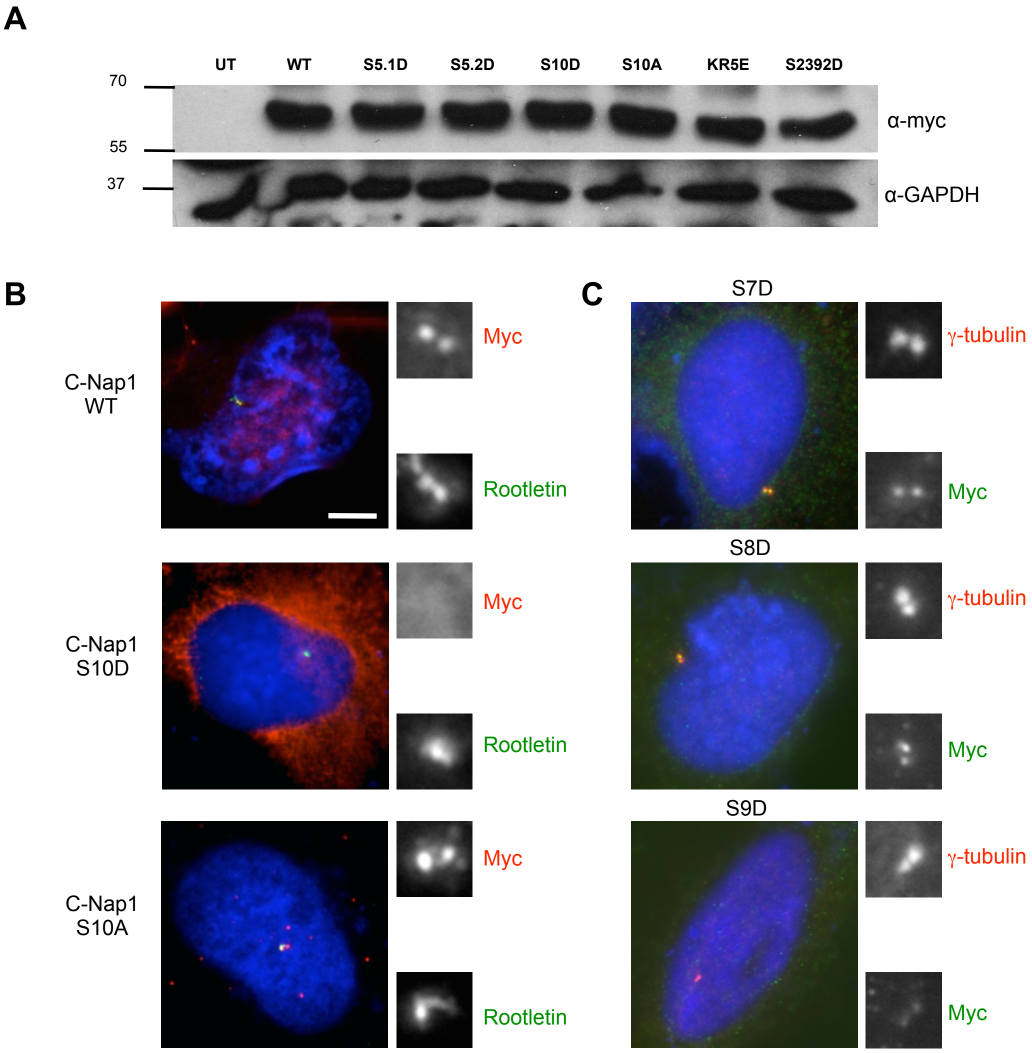
A
UT
WT
S5.1D
S5.2D
S10D
S10A
KR5E
S2392D
70
55
37
α-myc
α-GAPDH
B
C-Nap1
WT
Myc
Rootletin
C-Nap1
S10D
Myc
Rootletin
C-Nap1
S10A
Myc
Rootletin
C
S7D
γ-tubulin
Myc
S8D
γ-tubulin
Myc
S9D
γ-tubulin
Myc

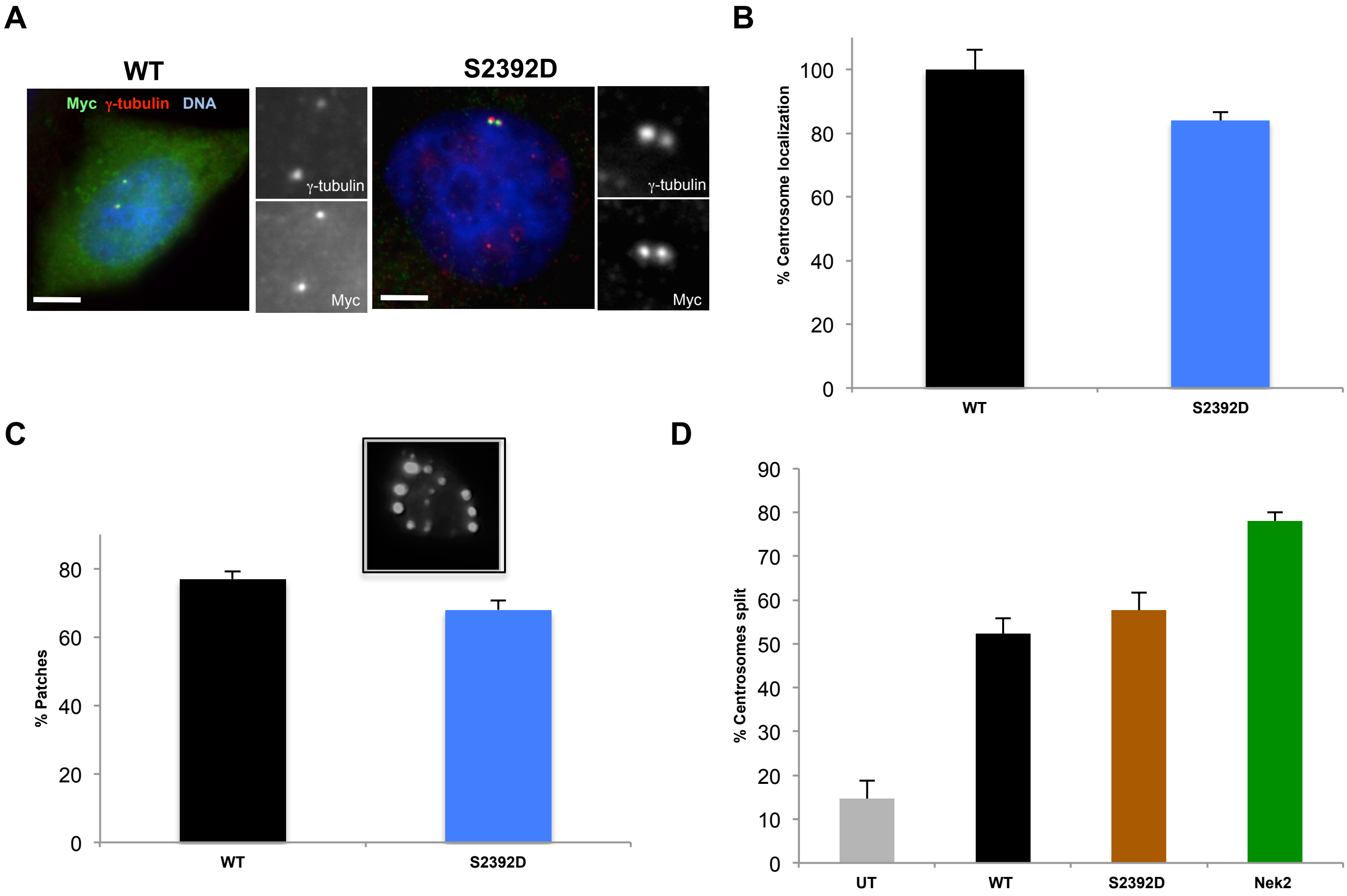
A
WT
S2392D
Myc γ-tubulin DNA
γ-tubulin
Myc
γ-tubulin
Myc
B
% Centrosome localization
100
80
60
40
20
0
WT
S2392D
C
% Patches
80
60
40
20
0
WT
S2392D
D
% Centrosomes split
90
80
70
60
50
40
30
20
10
0
UT
WT
S2392D
Nek2

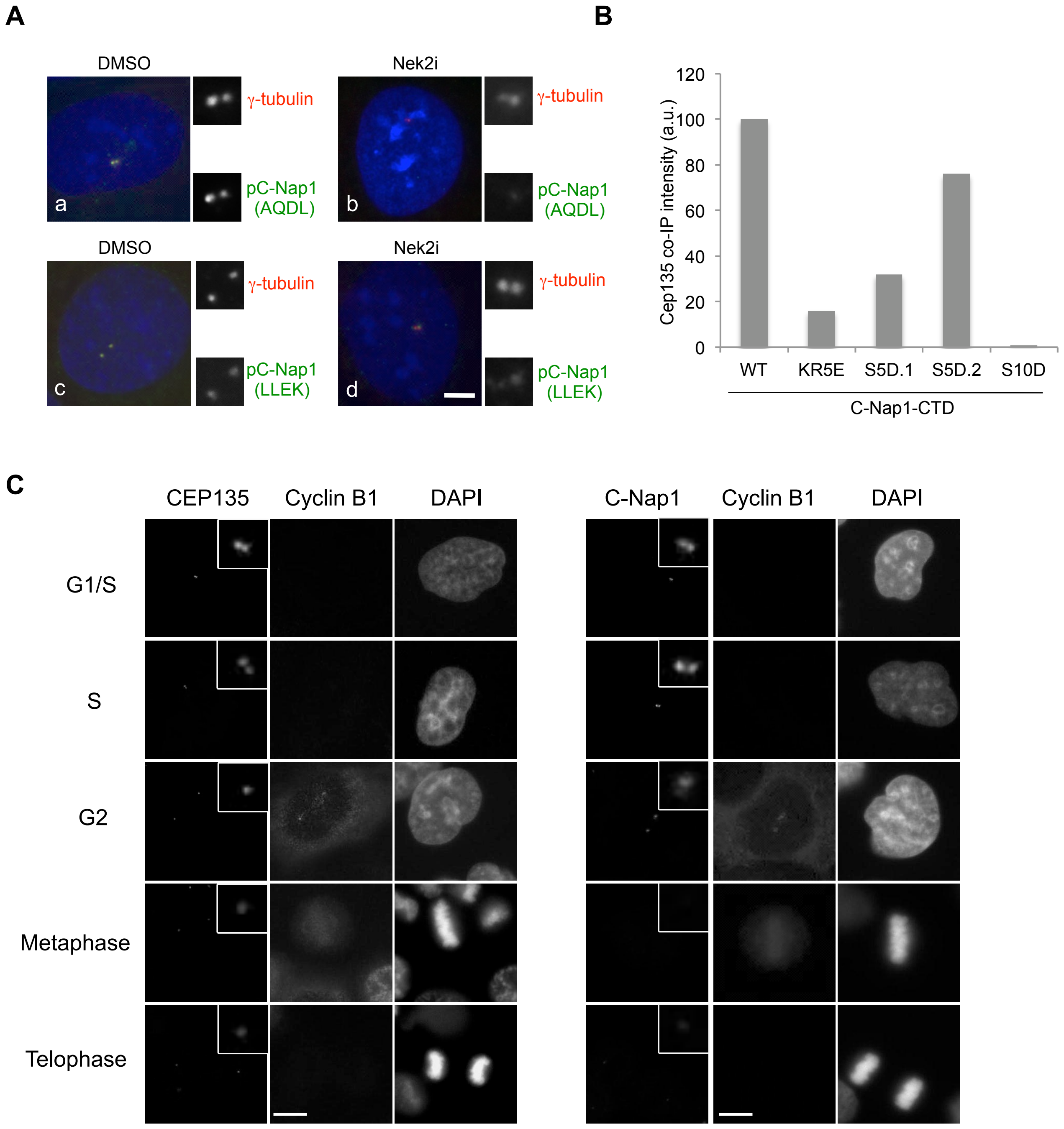
A
DMSO
γ-tubulin
pC-Nap1 (AQDL)
a
Nek2i
γ-tubulin
pC-Nap1 (AQDL)
b
DMSO
γ-tubulin
pC-Nap1 (LLEK)
c
Nek2i
γ-tubulin
pC-Nap1 (LLEK)
d
B
Cep135 co-IP intensity (a.u.)
0
20
40
60
80
100
120
WT
KR5E
S5D.1
S5D.2
S10D
C-Nap1-CTD
C
CEP135
Cyclin B1
DAPI
C-Nap1
Cyclin B1
DAPI
G1/S
S
G2
Metaphase
Telophase
